# Malaria Protein Kinase CK2 (PfCK2) Shows Novel Mechanisms of Regulation

**DOI:** 10.1371/journal.pone.0085391

**Published:** 2014-03-21

**Authors:** Michele Graciotti, Mahmood Alam, Lev Solyakov, Ralf Schmid, Glenn Burley, Andrew R. Bottrill, Christian Doerig, Paul Cullis, Andrew B. Tobin

**Affiliations:** 1 Department of Cell Physiology and Pharmacology, University of Leicester, Leicester, United Kingdom; 2 Medical Research Council Toxicology Unit, University of Leicester, Leicester, United Kingdom; 3 Department of Biochemistry, University of Leicester, Leicester, United Kingdom; 4 Department of Pure and Applied Chemistry, University of Strathclyde, Glasgow, United Kingdom; 5 The Protein Nucleic Acid Chemistry Laboratory, University of Leicester, Leicester, United Kingdom; 6 Department of Microbiology, School of Biomedical Sciences, Monash University, Clayton, Victoria, Australia; 7 Department of Chemistry, University of Leicester, Leicester, United Kingdom; Liverpool School of Tropical Medicine, United Kingdom

## Abstract

Casein kinase 2 (protein kinase CK2) is a conserved eukaryotic serine/theronine kinase with multiple substrates and roles in the regulation of cellular processes such as cellular stress, cell proliferation and apoptosis. Here we report a detailed analysis of the *Plasmodium falciparum* CK2, PfCK2, demonstrating that this kinase, like the mammalian orthologue, is a dual specificity kinase able to phosphorylate at both serine and tyrosine. However, unlike the human orthologue that is auto-phosphorylated on tyrosine within the activation loop, PfCK2 shows no activation loop auto-phosphorylation but rather is auto-phosphorylated at threonine 63 within subdomain I. Phosphorylation at this site in PfCK2 is shown here to regulate the intrinsic kinase activity of PfCK2. Furthermore, we generate an homology model of PfCK2 in complex with the known selective protein kinase CK2 inhibitor, quinalizarin, and in so doing identify key co-ordinating residues in the ATP binding pocket that could aid in designing selective inhibitors to PfCK2.

## Introduction

Despite the fact that protein kinases constitute an important target in the development of drugs to treat human disease [1], in particular cancer [2], progress in targeting protein kinases in malaria has been slow [3]. One reason for this is the paucity of basic understanding of parasite protein kinases. Bioinformatic studies have identified 85–99 protein kinases in the *Plasmodium falciparum* kinome. Although many of the *Plasmodium* protein kinases fall into established kinase groups such as AGC and CMGC (Hanks 2003), they tend to show significant diversity from mammalian species reflecting the fact that malaria parasites in the Apicomplexan phylum branched very early in the eukaryotic lineage [4], [5]. Despite this diversity it is possible to identify some *P. falciparum* protein kinases that do have counterparts in the mammalian kinome. One example of this is the *P. falciparum* orthologue to protein kinase CK2, PfCK2 [4], [5].

Mammalian protein kinase CK2 is a pleiotropic serine/threonine protein kinase know to act on hundreds of cellular substrates involved in crucial cellular processes such as differentiation, proliferation, apoptosis, stress response, DNA damage and circadian rhythm [6]. The mammalian enzyme is a tetramer consisting of two catalytic subunits, for which two different genes (α and α′) are present in the genome, and two regulatory β-subunits, derived from a single gene [7]. In the case of *P. falciparum* a single α-subunit catalytic gene has been identified and two genes encoding the regulatory subunits (β_1_ and β_2_) [8]. Reverse genetic studies have determined that not only the catalytic subunit for PfCK2, but also each of the regulatory β-subunits, are essential for the survival of *P. falciparum* through the erythrocytic life cycle [8] [9]. It seems likely that the essential nature of PfCK2 reflects a broad regulatory role similar to that of the mammalian orthologue. This is supported by the wide distribution of PfCK2 in the cytoplasm and nucleus of the parasite [9]. A more detailed analysis of the potential role of PfCK2 in the nucleus has identified a number of interacting partners and substrate nuclear proteins involved in chromatin assembly and dynamics [9].

Here we extend these early studies and define that PfCK2 is a dual specificity protein kinase able to phosphorylate both serine and tyrosine residues. Furthermore, we demonstrate that PfCK2 undergoes auto-phosphorylation and that this event regulates kinase activity, albeit through a mechanism that diverges from that operating with the mammalian CK2. We thus demonstrate features of PfCK2 that are similar to those of its mammalian orthologue and features that are specific to *P. falciparum*.

## Experimental Procedures

### Materials

Human casein kinase II, 10× CK2 kinase buffer and CK2 peptide substrate RRRADDSDDDDD were purchased from New England Biolabs and ZipTip from Millipore (Waltford, UK). Quinalizarin was purchased from CHEMOS GmbH (Regenstauf, Germany), Radioisotope [^32^P]-ATP (specific activity 3000Ci/mmol) was purchased from Perkin Elmer Life Science. For GST fusion protein purification, Amintra glutathione resin was purchased from Expedeon (Haxton, UK).

### Expression and purification of the GST-proteins

Synthetic DNA encoding for the PfMCM2 1–159 protein fragment from *P. falciparum* (clone3D7A) carring BamI 5′end and EcoRI 3′ end restrinction sites was provided by Eurofins MWG Operon (Ebersberg, Germany). The cDNA was inserted into pGEX-4T3 vector (GE Healthcare) to generate N-terminal glutathione S-transferase (GST) fusions. GST-MCM2 mutants were obtained by site-directed mutagenesis by overlap extension PCR using the following primers: for S13A mutant: Forward: 5′-GAAGATCTGGAAGCCAACAAATATGATATTG-3′, Reverse: 5′-CAATATCATATTTGTTGGCTTCCAGATCTTC-3′; for Y16A mutant: Forward: 5′-CTGGAAAAGCAACAAATTCGATATTGATGAAGAAGATCTGCTGG-3′, Reverse: 5′-CCAGCAGATCTTCTTCATCAATATCGAATTTGTTGCTTTCCAG-3′; for S13A-Y16F double mutant: Forward: 5′-GAAGATCTGGAAGCCAACAAATTCGATATTG-3′, Reverse: 5′-CAATATCGAATTTGTTGGCTTCCAGATCTTC-3′. A pGEX-4T3 vector containing *P. falciparum* CK2 in frame with an N-terminal GST tag was kindly provided by Prof. Christian Doerig. The pGEX4T3 constructs were expressed in *Escherichia coli* BL21 cells for 20 h at 20°C with 0.1 mM isopropyl-D-thiogalactopyranoside (IPTG). GST-tagged proteins were purified on glutathione-agarose beads, following the manufacturer's recommendations.

### *In vitro* PfCK2 protein kinase assay

Standard kinase reactions (25 µl) occurred in kinase buffer (20 mM Tris-HCl [pH 7.5], 20 mM MgCl_2_, 50 mM KCl) containing 50 µM [γ-^32^P]-ATP (500–1000 c.p.m./pmol), 1 µg of recombinant kinase, and substrate (5 µg). Reactions were carried out at 37°C for 15 min and terminated by the addition of Laemmli buffer. Samples were separated by sodium dodecyl sulfate-polyacrylamide gel electrophoresis (SDS-PAGE). The gels were dried and exposed for autoradiography. For PfCK2 autophosphorylation assay reactions were carried out following the same protocol in the absence of substrate. For quantification gel bands were cut and quantified by scintillation counting. For LC-MS/MS analysis the reaction was carried out in the absence of [γ-^32^P]-ATP, pellets were run on an SDS-gel and bands cut and analysed as reported below.

### Phospho-proteomic LC-MS/MS analysis of tryptic peptides

Bands of interest were excised and in-gel trypsin digestion carried out. The digests were then analysed by LC-MS/MS using an LTQ Orbitrap-Velos mass spectrometer (Thermo Scientific). Samples were loaded at high flow rate onto a reverse-phase trap column (0.3 mm i.d.×1 mm), containing 5 µm C18 300 Å Acclaim PepMap media (Dionex) maintained at a temperature of 37°C. The loading buffer was 0.1% formic acid/0.05% trifluoroacetic acid in water. Peptides were eluted from the trap column at a flow rate of 0.3 µl/min and through a reverse-phase capillary column (75 µm i.d.×250 mm) containing Symmetry C18 100 Å media (Waters, UK) that was manufactured in-house using a high pressure packing device (Proxeon Biosystems, Denmark). The output from the column was sprayed directly into the nanospray ion source of the LTQ-Orbitrap-Velos mass spectrometer. The LTQ-Orbitrap-Velos mass spectrometer was set to acquire a 2 microscan FTMS scan event at 30000 resolution over the m/z range 400–1800 Da in positive ion mode. Obtained data were processed with Mascot (version 2.2.04, Matrix Science Ltd., UK) and Scaffold (version 4.0.5, Proteome Software).

### PfCK2 inhibition assay

To test the effect of quinalizarin on PfCK2, kinase activity was measured in the presence of increasing concentrations of this molecule, stocks solutions were prepared in dimethyl sulfoxide, and negative controls for the reactions contained dimethyl sulfoxide without the small molecule inhibitor. Kinase reactions were performed by the phosphocellulose method. Briefly, CK2 activity was tested in a final volume of 25 µl containing 20 mM Tris/HCl (pH 7.5), 50 mM KCl, 10 mM MgCl_2_, 200 µM synthetic peptide substrate RRRADDSDDDDD and 0.1 mM [γ-^32^P]-ATP (500–1000 c.p.m./pmol), 0.5 µg enzyme, and incubated for 10 min at 37°C. Assays were stopped by addition of 5 µl of 0.5M orthophosphoric acid before spotting 15 µl aliquots on to phospho-cellulose filters. Filters were washed in 0.05%orthophosphoric acid (5–10 ml each) four times then once in methanol and dried before scintillation counting.

### *In silico* homology model of PfCK2

Amino acid sequences of CK2 catalytic subunits from Homo sapiens (CAB65624), and *Plasmodium falciparum* (AAN35684; PF3D7_1108400) and other CK2 orthologs were aligned using ClustalW [10] and adjusted manually. A series of homology models for the *P. falciparum* CK2 catalytic subunit was built in Modeller 9.10 [11] using the human CK2 crystal structure (PDB code: 1JWH) [12] and the CK2 structure from *Zea mays* (PDB code: 3FL5) as templates [13]. Alternative models which include a structurally conserved water molecule relevant for the inhibitor binding mode were built following the same procedure.

### *In silco* docking simulation of quinalizarin with PfCK2

A docking simulation with the inhibitor quinalizarin was performed with the calculated homology model for *P. falciparum* CK2, using the programme GOLD116 and GoldScore as a scoring function. Hydrogen atoms were introduced in the protein structure with the default GOLD command and in the ligand molecule with the programme HyperChem. To strictly validate the model generated and to calibrate the docking protocol, a docking analysis was first performed using the apo structure of the co-crystal model of CK2 (PDB code: 3FL5), with the CK2 inhibitor quinalizarin. Superimposition images were obained using Pymol.

## Results

### PfCK2 is a dual specificity kinase

**Figure 1:**
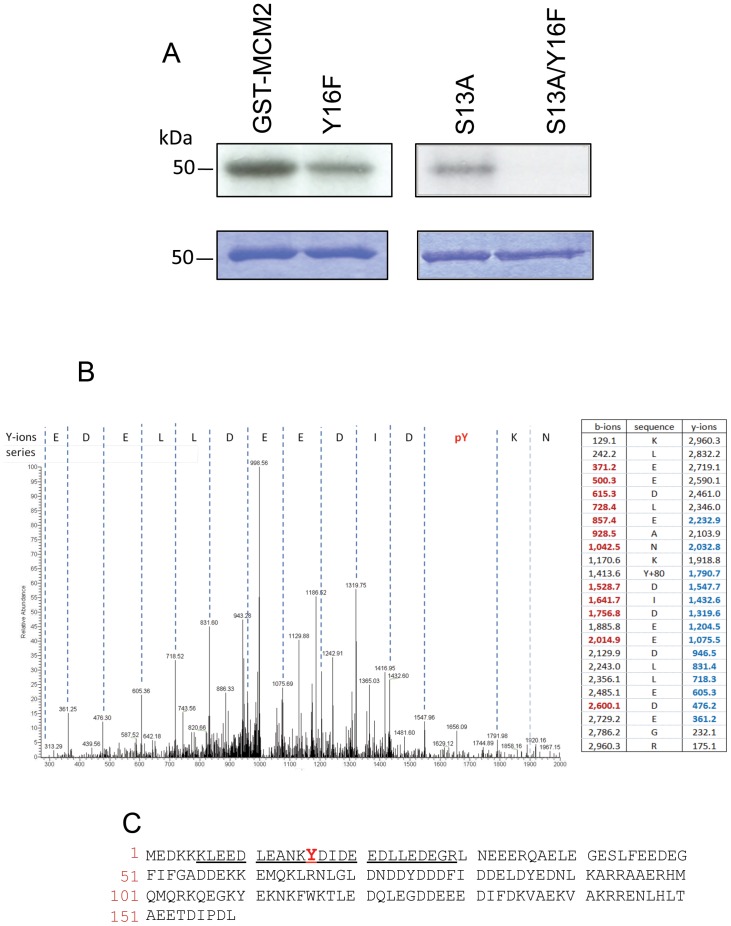
PfCK2 phosphorylates MCM2 on Ser13 and Tyr16 *in vitro*. **A**: In vitro kinase assay using a GST fusion protein containing a N-terminal portion of MCM2 (GST-MCM2) or the same fusion protein but where residue Y16 is mutated to an phenylalanine (Y16F) or where residue S13 is mutated to an alanine (S13A) or where both S13 and Y16 are mutated to an alanine and phenylalanine respectively (S13A/Y16F). Top panel: autoradiograph, bottom panel: Coomassie stain. **B**: LC-MS/MS trace of the fusion protein GST-PfMCM2 containing the S13 to alanine mutation following phosphorylation with PfCK2 indicating the phosphorylation of residue Y16. Also shown is the fragmentation table (detected b-ions and y-ions are represented respectively in bold red and bold blue). **C**: N-terminal sequence of PfMCM2 protein showing the phospho-peptide identified in the LC-MS/MS analysis that contains the tyrosine phosphorylated residue (in red).

Bioinformatic analysis of the *P. falciparum* kinome revealed there are no member of the tyrosine kinase (TK) group in the parasite genome [4], [5]. Despite this, phosphoproteome studies [14]–[16] have demonstrated that parasite tyrosine phosphorylation can be detected. This derives in part from the activity of protein kinases such as PfCLK3 and PfGSK3, where auto-phosphorylation on tyrosine is an intrinsic mechanism of kinase activation [15]. However, tyrosine phosphorylation can also be detected on proteins that appear to be substrates for parasite protein kinases that can phosphorylate on tyrosine [14]–[16]. One such putative substrate protein is the minichromosome maintenance (MCM) complex subunit, PfMCM2 (PF3D7_1417800), a protein likely to be involved in the initiation of genome replication [15]. Our analysis, and that of others, have indicated that PfMCM2 is phosphorylated *in vivo* at serine 13 (S^13^) and possibly on tyrosine 16 (Y^16^) [15], [16]. These residues are within an acidic region that resembles the consensus sequence for protein kinase CK2 ((S/T-x-x-E/D/pS) [6]. Thus, we tested if *P. falciparum* PfCK2 (PF3D7_1108400) could phosphorylate S^13^ and/or Y^16^ on PfMCM2 by conducting an *in vitro* kinase assay using recombinant PfCK2 α-subunit [8] and a glutathione-s-transferase:PfMCM2 fusion protein (GST-MCM2) containing the first 159 amino acids of PfMCM2 as a substrate (Figure 1A). The GST-MCM2 fusion protein acted as a substrate for PfCK2 α-subunit (Figure 1A). Mutation of either S^13^ or Y^16^ on the PfMCM2 portion reduced the phosphorylation status of the GST-MCM2 fusion protein whereas the removal of both of these residues eliminated phosphorylation completely (Figure 1A), indicating that PfCK2 α-subunit could phosphorylate both at serine-13 and tyrosine-16 within the GST-MCM2 fusion protein. The ability of PfCK2 α-subunit to act as a tyrosine kinase in this *in vitro* reaction was confirmed by mass spectrometry analysis that identified PfCK2-mediated Y^16^ phosphorylation of a GST-MCM2 fusion protein (Figure 1B,C).

### Characterisation of the auto-phosphorylation of PfCK2

**Figure 2:**
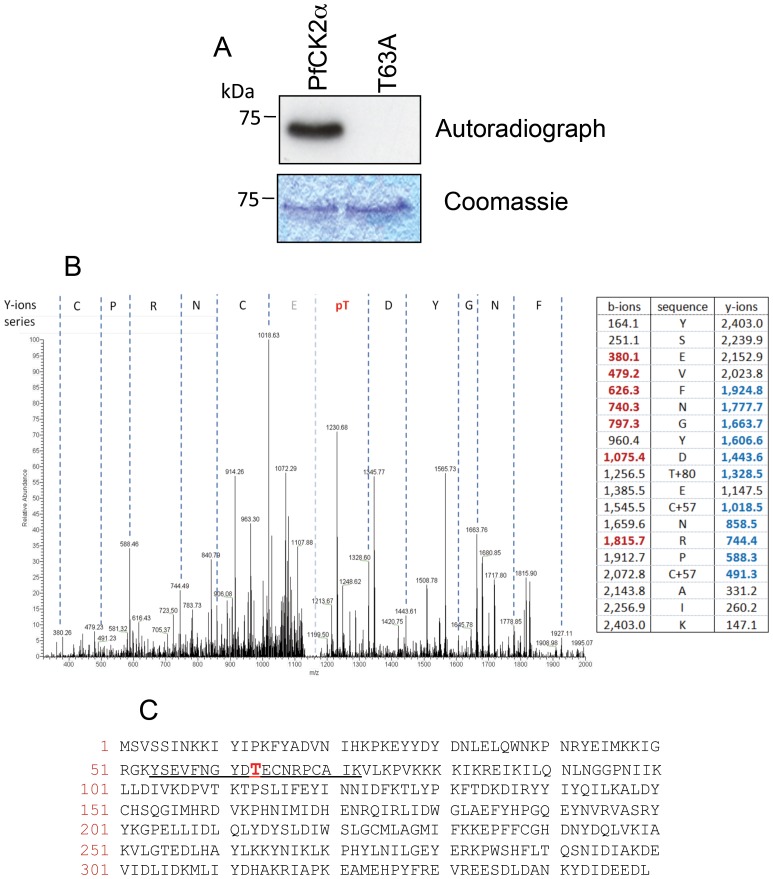
PfCK2 auto-phosphorylates *in vitro* on threonine 63. **A**: *In vitro* kinase assay for GST-PfCK2 autophosphorylation, top panel: autoradiograph, bottom panel: Coomassie stain. **B**: LC-MS/MS trace identifying phosphorylation of PfCK2 at T63; right: Also shown is the hypothetical fragmentation table where the b-ions and y-ions detected in the LC-MS/MS spectra are shown in red and bold, respectively. **C**: Sequence of PfCK2 showing the phosphopeptide identified in the LC-MS/MS analysis (underlined) and the threonine 63 phosphorylation site (in red).

Purified recombinant PfCK2 α-subunit was seen to undergo auto-phosphorylation (Figure 2A), as previously reported [Holland 2009]. Mass spectrometry analysis determined that threonine 63 was the site of auto-phosphorylation (Figure 2B). Mutation of threonine 63 to alanine abolished auto-phosphorylation (Figure 2A), indicating that this residue is likely to be the only site of auto-phosphorylation. This is important since previous studies on human protein kinase CK2α had reported auto-phosphorylated on tyrosine 182 in the activation loop of the kinase [17]. Despite the fact that this activation loop tyrosine is conserved in PfCK2 (tyrosine 189) we could not detect any evidence of auto-phosphorylation at this residue. This is illustrated not only by the fact that all auto-phosphorylation was lost following mutation of threonine 63 to alanine in PfCK2, but also by the fact that mass spectrometry analysis identified the peptide LIDWGLAEF**Y**HPGQEYNVR (Y^189^ underlined and bold) but this peptide was observed only in the non-phosphorylated state.

**Figure 3:**
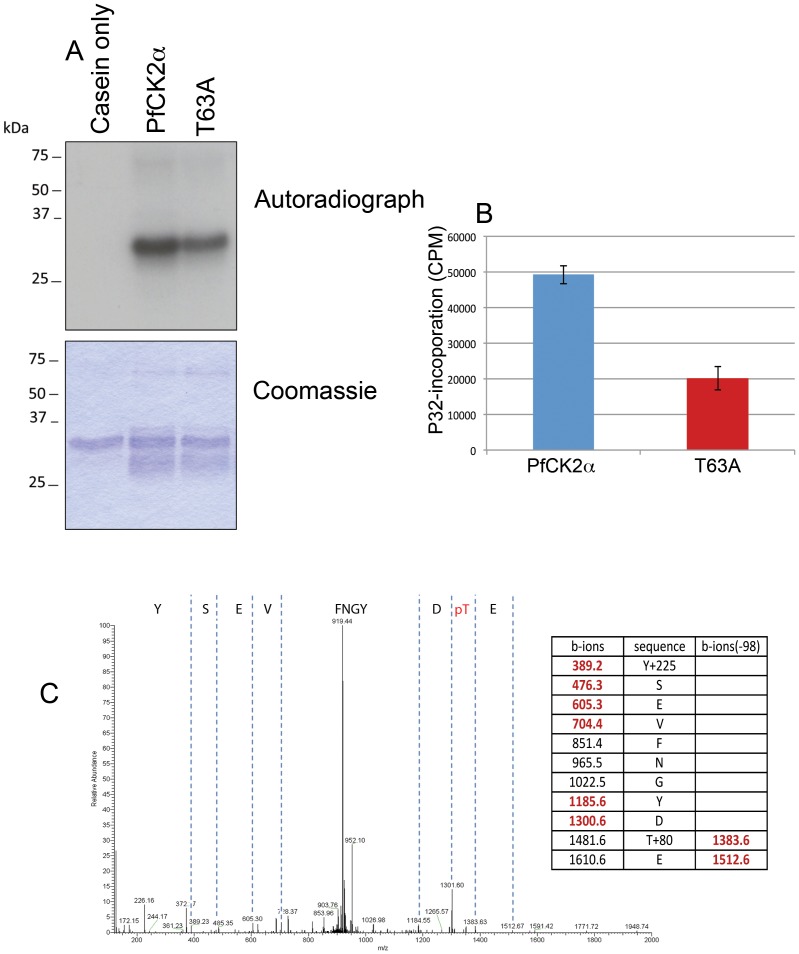
Autophosphorylation of PfCK2 regulates kinase activity. The activity of PfCK2α and a mutant PfCK2α where threonine 63 was mutated to alanine (T63A) was tested in *in vitro* kinase assays using α-casein as a substrate. **A**: Example of the *in vitro* kinase assay with PfCK2α and the T63A mutant. Top panel: autoradiograph, bottom panel: Coomassie stain. **B**: kinase activity quantification. Date represents the mean ± S.E.M (n = 3) **C**: LC-MS/MS trace of PfCK2 identifying T63 phosphorylation from a shizont stage lysate of *P. falciparum*. Indicated are the b-ions and b-ions (−98daltons) that were identified in the LC-MS/MS spectra. Also shown is the hypothetical fragmentation table where the ions that were identified in the LC-MS/MS spectra are shown in red.

These studies established T^63^ in sub-domain I of the PfCK2α as the site of auto-phosphorylation. Importantly, mutation of this site to an alanine not only abolished auto-phosphorylation, but also reduced the kinase activity of PfCK2 α-subunit on exogenous substrate by ∼60% (Figure 3A,B). Furthermore, recent analysis of the phospho-proteome of the schizont stage of *P. falciparum* identified a phosphorylation of T^63^ in the α-subunit of PfCK2, demonstrating that this phosphorylation event occurs in vivo (Figure 3C).

### Characterisation of the inhibition of PfCK2 by quinalizarin

**Figure 4:**
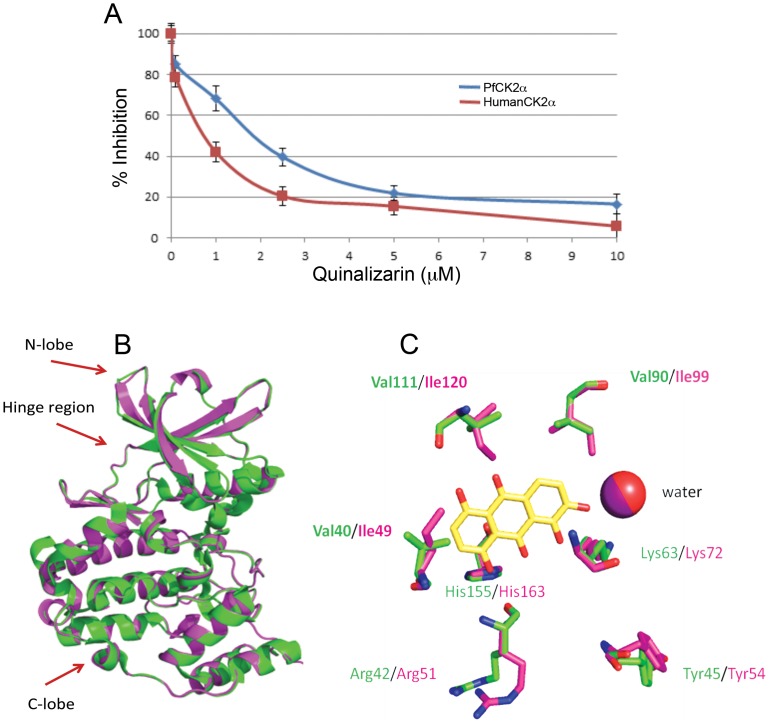
Structural analysis of PfCK2α inhibition by quinalizarin. **A**: *In vitro* inhibition assay showing the affect of various concentrations of quinalizarin on the activity of human protein kinase CK2α (red) and PfCK2α (blue). Date represents the mean ± S.E.M (n = 3) **B**: Superimposition of the calculate *in silico* homology model for PfCK2α (purple) with the *Zea mays* protein kinase CK2α crystal structure (green, PDB code: 3FL5, resolution 2.30 Å); **B**: Superimposition of the molecular docking of the PfCK2 homology model with quinalizarin (purple) and the co-crystal structure of *Z. mays* protein kinase CK2α and quinalizarin (green); non-conserved residues are indicated in bold.

To improve our understanding of PfCK2 and to gain important structural information that might guide the design of new parasite kinase inhibitors, we decided to test quinalizarin, the most potent and selective inhibitor of human protein kinase CK2 commercially available [13], in *in* vitro kinase assays with PfCK2. We found that quinalizarin inhibited both parasite and human CK2 with similar potency and efficacy, where the IC_50_ values were 2 µM and 0.8 µM respectively (Figure 4A). These data suggested that quinalizarin interacted with the ATP binding pocket of human and *P. falciparum* protein kinase CK2 similarly. To investigate this further we created an *in silico* model of quinalizarin in complex with PfCK2. In order to do this we based the *in silico* structure of the PfCK2 α-subunit on the reported crystal structure of *Zea mays* protein kinase CK2 (65% sequence identity to PfCK2) in complex with quinalizarin (PDB code 3FL5, resolution 2.3 Å). Figure 4B shows the superimposition of the crystal structure of protein kinase CK2 from *Z. mays* with the *in silico* model of PfCK2. In these structures the binding mode for quinalizarin in the ATP binding pocket of protein kinase CK2 from *Z. mays* and *P. falciparum* is essentially identical (Figure 4C). Interestingly, not all of the residues forming the binding site are fully conserved. Residues V^40^, V^90^ and V^111^ in *Z. mays* protein kinase CK2 correspond to more bulky isoleucine residues in PfCK2 (Figure 4C). In human protein kinase CK2 the corresponding residues are leucine, valine and isoleucine, respectively. This suggests that the binding site for quinalizarin in the ATP binding pocket of protein kinase CK2 from the three species investigated is subtly different, with PfCK2α being the most spatially restricted.

## Discussion

Recent kinome wide analysis of the protein kinases in the asexual blood stage of *P. falciparum* [15] and sexual/insect stages of *Plasmodium berghei* [18] have identified essential protein kinases throughout the parasite life cycle as validated targets for drug discovery aimed at both curative treatments and transmission blockers [3], [19], [20]. One of the barriers to pursuing these protein kinase targets is the paucity of basic information regarding the fundamental mechanisms of action of the essential parasite kinases. We address this issue here by focusing on PfCK2, a close orthologue to the mammalian protein kinase CK2, which is known to be essential for the blood stage of the parasite [8], [9]. We show that PfCK2, like its mammalian orthologue, is capable of catalyzing both tyrosine and serine phosphorylation, and this at least partly explains the presence of tyrosine-phosphorylated proteins (such as MCM2) in the parasite phosphoproteome. However, unlike mammalian protein kinase CK2, PfCK2 is not autophosphorylated on a conserved tyrosine within the activation loop but rather is autophosphorylated on a unique threonine in domain I (T63) of the catalytic subdomain in a manner that regulates PfCK2 enzymatic activity.

The tyrosine kinase group of eukaryotic protein kinases is generally thought to have evolved as a metazoan-specific family to meet the demands of development, differentiation and intercellular communication [21]. Hence, worms (e.g. *Caenorhabditis elegans*), flies (e.g. *Drosophilia melanogaster*) and mammals have extensive tyrosine kinase groups [21]. In contrast, although there are some rare exceptions such as the Choanoflagellates, a group of aquatic flagellate unicellular eukaryotes considered to be the closest relatives to the animals (metazoans), which have an extensive tyrosine kinase family [22], [23] generally protozoans such as yeast (Saccharomyces cerevisiae) [21] and human parasites including *Trypanosoma brucei* (causative agent of sleeping sickness) [24], [25] and *Giardia lamblia* (human gut parasite) [26] as well as parasites of the Apicomplexa phylum including *Toxoplasma gondii* [27] and *P. falciparum* [4], [5] do not contain any recognizable member of the tyrosine kinase group. Despite the absence of members of the TK group, many protozan phospho-proteomes have been reported to contain proteins phosphorylated on tyrosine residues. This includes the phospho-proteomes of yeast [28], *Trypanosoma brucei* [*24*], *Giardia lamblia* [*26*], [*29*], *Toxoplasma gondii* and *P. falciparum* [14]–[16], [30], [31]. Importantly, in *P. falciparum* the tyrosine phosphorylation sites detected with the highest confidence are those associated with kinase auto-phosphorylation [15], [32] particularly of kinases that behave as DYRKs, such as PfCLK3 [15], [30], [33], where tyrosine phosphorylation is an important process during translation, occurring at translational intermediates, after which the mature kinases have serine/threonine kinase activity [34]–[36]. Although rare, there are tyrosine phosphorylated proteins other than kinases which have undergone autophosphorylation. That this is the case in *P. falciparum* is evident in phospho-proteomic [15], [30], [33], western blot [31], pharmacological [30], [37], [38] and biochemical studies [15] and points to the possibility that there exist dual specificity kinases able to trans-phosphorylate on tyrosine as well as serine/threonine residues. The finding here that PfCK2, like its mammalian homologue, has dual specificity kinase activity *in vitro* suggests that PfCK2 might contribute to tyrosine phosphorylation in the malaria parasite.

Although PfCK2 shares the characteristic of having dual kinase specificity with its mammalian orthologue, the parasite kinase appears to be regulated in a unique manner. Mammalian protein kinase CK2 is autophosphorylated in a *trans* intermolecular mechanism on tyrosine in the activation loop, and this appears to regulate the enzymatic activity [17]. Despite PfCK2α possessing a tyrosine in an analogous position, this autophosphorylation has not been detected either in vivo or with the recombinant enzyme, in this study or in the previously published data bases [14]–[16]. It is possible that the mass spectrometry approaches used here were not sensitive enough to detect this phosphorylation event and that more focused approaches, such as Multiple Reaction Monitoring or Selected Reaction Monitoring, might have detected this phosphorylation event. What is certain, however, is that PfCK2 is autophosphorylated on threonine 63 in subdomain I of the catalytic domain. Mutation of this site to alanine results in decreased enzymatic activity, indicating that this may be a mechanism of regulating PfCK2 activity *in vivo*. This is the first report of threonine 63 phosphorylation on PfCK2α [14]–[16] and importantly this phosphorylation event which we first detected *in vitro* is also seen our most recent *in vivo* global phosphorylation analysis.

The role of human protein kinase CK2 in the regulation of cell proliferation and apoptosis, together with the fact that this kinase is up-regulated in cancer, has led to efforts to target protein kinase CK2 in the treatment of cancer [39], [40]. Generating selective inhibitors with high potency and drug-like pharmacokinetic properties has however been challenging (see for examples refs [41], [42]), although a protein kinase CK2 inhibitor, CX-4945, has been tested in clinical trials for the treatment of cancer [43], [44]. Any anti-malarial treatment based on the inhibition of parasite PfCK2 would have to be highly specific for PfCK2, as interaction with the human orthologue would likely cause serious adverse affects. Thus, here we made an initial analysis of the binding mode of a well characterized inhibitor of protein kinase CK2, quinalizarin [13] to PfCK2. We based our homology model on the available crystal structure of quinalizarin in complex with the α-subunit of protein kinase CK2 from *Z. mays*. . Our analysis suggests that the ATP binding pocket of PfCK2, *Z. mays* and human are very similar to each other. This is reflected in the fact that quinalizarin has very similar inhibitor properties between PfCK2 and human protein kinase CK2. However, three residues of the binding pocket are overall more bulky in the case of PfCK2, suggesting that the inhibitor binding pocket is more restricted in PfCK2 than is the case for protein kinase CK2 from *Z. mays* and from human. Whether these subtle differences are sufficient to be exploited in the development of novel, selective, inhibitors to PfCK2 is currently being considered.
